# Pharmacokinetics of rivaroxaban in children using physiologically based and population pharmacokinetic modelling: an EINSTEIN-Jr phase I study

**DOI:** 10.1186/s12959-018-0185-1

**Published:** 2018-12-04

**Authors:** Stefan Willmann, Kirstin Thelen, Dagmar Kubitza, Anthonie W. A. Lensing, Matthias Frede, Katrin Coboeken, Jan Stampfuss, Rolf Burghaus, Wolfgang Mück, Jörg Lippert

**Affiliations:** 1 Clinical Sciences, Bayer AG, Bayer AG, Aprather Weg 18a, Wuppertal, Germany; 2 Clinical Development, Bayer AG, Wuppertal, Germany; 3 Clinical Pharmacometrics, Bayer AG, Leverkusen, Germany; 4 Clinical Pharmacology, Bayer AG, Wuppertal, Germany

**Keywords:** Pediatric, Pharmacokinetics, Physiologically based pharmacokinetic modelling, Rivaroxaban

## Abstract

**Background:**

The EINSTEIN-Jr program will evaluate rivaroxaban for the treatment of venous thromboembolism (VTE) in children, targeting exposures similar to the 20 mg once-daily dose for adults. A physiologically based pharmacokinetic (PBPK) model for pediatric rivaroxaban dosing has been constructed.

**Methods:**

We quantitatively assessed the pharmacokinetics (PK) of a single rivaroxaban dose in children using population pharmacokinetic (PopPK) modelling and assessed the applicability of the PBPK model. Plasma concentration–time data from the EINSTEIN-Jr phase I study were analysed by non-compartmental and PopPK analyses and compared with the predictions of the PBPK model. Two rivaroxaban dose levels, equivalent to adult doses of rivaroxaban 10 mg and 20 mg, and two different formulations (tablet and oral suspension) were tested in children aged 0.5–18 years who had completed treatment for VTE.

**Results:**

PK data from 59 children were obtained. The observed plasma concentration–time profiles in all subjects were mostly within the 90% prediction interval, irrespective of dose or formulation. The PopPK estimates and non-compartmental analysis-derived PK parameters (in children aged ≥6 years) were in good agreement with the PBPK model predictions.

**Conclusions:**

These results confirmed the applicability of the rivaroxaban pediatric PBPK model in the pediatric population aged 0.5–18 years, which in combination with the PopPK model, will be further used to guide dose selection for the treatment of VTE with rivaroxaban in EINSTEIN-Jr phase II and III studies.

**Trial registration:**

ClinicalTrials.gov number, NCT01145859; registration date: 17 June 2010.

## Introduction

International guidelines recommend the use of unfractionated heparin, low molecular weight heparin and vitamin K antagonists for the treatment of venous thromboembolism (VTE) in children [1]. However, because of a lack of robust clinical evidence, treatment recommendations are largely extrapolated from adult data [2–7].

Physiologically based pharmacokinetic (PBPK) modelling is increasingly being used as a tool to guide dosing in children [8–11]. PBPK models, based on actual organs with their inherent volumes and blood flows linked through the vasculature [12], and defined processes of absorption, distribution, metabolism and excretion as a function of anatomy, physiology and biochemistry, permit rational scaling between species and developmental stages [8].

A pediatric PBPK model of rivaroxaban has been developed [13], which considers a priori known age-specific physiological changes that affect the pharmacokinetics (PK) of rivaroxaban, such as the ontogeny of relevant hepatic and renal elimination processes, and age effects on absorption. During the pediatric development of rivaroxaban, this model provided the basis for the selection of the first-in-children doses in accordance with the ‘predict-learn-confirm’ paradigm [8].

Rivaroxaban is approved in adults for the treatment of VTE [14, 15], but data in children are lacking. Ex vivo spiking experiments suggested that the response to exposure to Factor Xa inhibition with rivaroxaban in children may be similar to that in adults [16, 17]. The EINSTEIN-Jr program will evaluate rivaroxaban for the treatment of VTE, aiming for a rivaroxaban exposure in children similar to the exposure observed in adult patients with VTE who have received rivaroxaban 20 mg.

In this study, we present the PK data from the EINSTEIN-Jr phase I study, which evaluated a single rivaroxaban administration in children aged 0.5–18 years.

## Methods

### Participants and study design

EINSTEIN-Jr phase I (NCT01145859) was a multinational, multicentre, single-dose study in children aged 0.5–18 years who had completed treatment for VTE. Study design and eligibility criteria are detailed in an accompanying report [18]. The study was approved by the Institutional Review Board at each study site. The parent or legal guardian provided written, informed consent and the child signed the assent form, if applicable. An independent Data Monitoring Committee periodically evaluated safety and PK/pharmacodynamic (PD) data. The study was conducted in accordance with the Declaration of Helsinki.

**Fig. 1:**
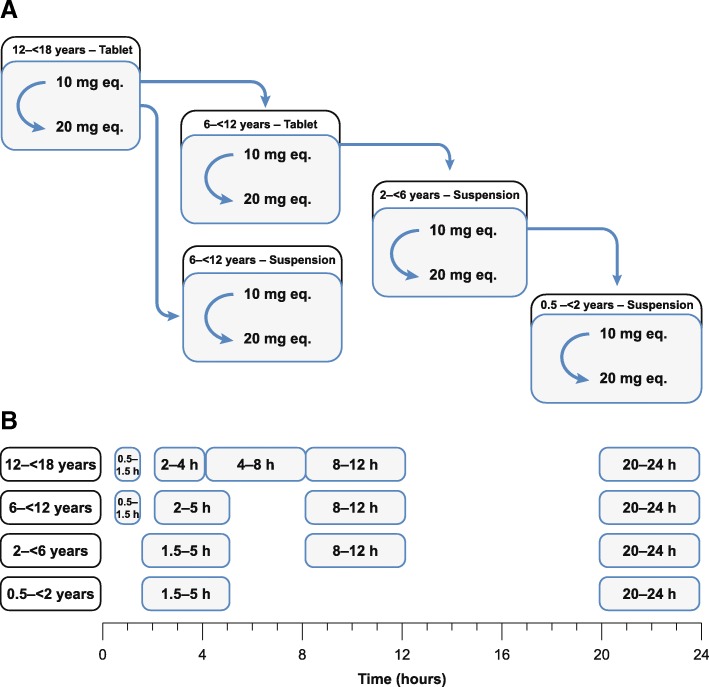
**a** Staggered approach and sampling strategy for collecting PK/PD and safety data in pediatric cohorts in this phase I study. PK/PD and safety data available for at least four subjects before starting next cohort; blue arrows indicate data monitoring committee agreement to progress to the next planned step of the phase I study, i.e. data collection for the next cohort. **b** Sampling windows per age group eq., equivalent, *PD* pharmacodynamics, *PK* pharmacokinetics.

Subjects received a body-weight-adjusted single dose of rivaroxaban within 2 h of a meal, with the selected dose aiming to achieve similar exposure to that observed in adults receiving rivaroxaban 10 mg or 20 mg. We evaluated: four age groups (0.5–< 2, 2–< 6, 6–< 12 and 12–< 18 years), two dose groups (rivaroxaban 10 mg and 20 mg-equivalent) and two formulation groups (tablet or oral suspension) (Fig. 1). Children aged ≥12 years received rivaroxaban as a tablet, children aged 6–< 12 years received rivaroxaban as a tablet or oral suspension at the discretion of the investigator, and children aged < 6 years received rivaroxaban as an oral suspension. The oral suspension was administered either as an undiluted or diluted suspension. The study was conducted in a staggered fashion by age group and dose, starting with children aged 12–< 18 years receiving the 10 mg-equivalent dose. Sequential steps were taken through the decision tree for the other cohorts.

PK blood sampling was carried out at different time points after study drug administration depending on the age group: at 90 min–5 h and 20–24 h in children aged 0.5–2 years and 2–6 years, with an additional measurement at 8–12 h in those aged 2–6 years. In children aged 6–12 years and 12–18 years, measurements were taken at 30–90 min, and 2–5, 8–12 and 20–24 h, with an additional measurement at 4–8 h in those aged 12-18 years (Fig. 1). Blood samples were collected by venipuncture, central venous line or peripheral catheter, stored at or below − 15 °C and centrally analysed within 4 weeks. Rivaroxaban plasma concentrations were determined using high-performance liquid chromatography–tandem mass spectrometry detection after solid/liquid extraction [19]. The calibration range of the procedure was from 0.500 μg l^− 1^ (lower limit of quantification [LLOQ]) to 500 μg l^− 1^ (upper limit of quantification). Mean inter-assay accuracy of back-calculated concentrations (except LLOQ) in calibrators ranged between 93.3 and 104.5% and precision was ≤4.4%. Rivaroxaban plasma concentrations were calculated from the chromatographic raw data.

### Modelling strategy

**Fig. 2:**
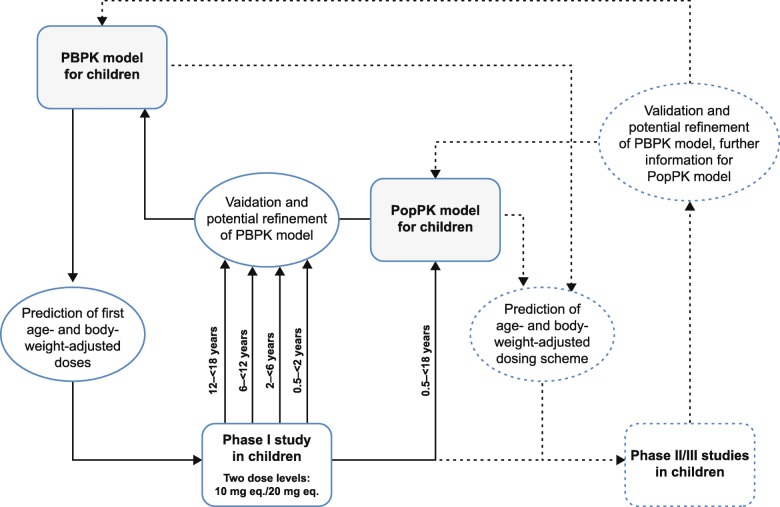
Schematic diagram of the integration of modelling and simulation approaches into the pediatric development process of rivaroxaban. Dashed arrows denote work in progress; outcomes of this work will be described in future publications eq., equivalent; PBPK, physiologically based pharmacokinetic; PopPK, population pharmacokinetic.

Figure 2 illustrates the workflow of how PBPK and population PK (PopPK) modelling were employed throughout the pediatric development program of rivaroxaban. The main goal of the modelling activities was to have valid and predictive PBPK and PopPK models that can be applied to define dosing regimens for the EINSTEIN-Jr phase II and III studies.

The pediatric PBPK model delivered predictions of rivaroxaban exposure for two dose levels (rivaroxaban 10 mg per 70 kg and 20 mg per 70 kg) in different age groups (‘prediction’ step) [13]. Based on these predictions, a first body-weight-adjusted dose was tested in the first-in-children study (‘learning’ step). The doses were selected in a way that the resulting exposure would be similar to the exposure observed in healthy adults who received either rivaroxaban 10 mg or 20 mg. After completion of each age cohort, PK data were compared with the model predictions to allow for PBPK model refinement. After the finalization of all age cohorts, a first PopPK model of rivaroxaban in children was developed, which was applied to estimate post hoc PK parameters for each individual child that were later on compared with the PBPK predictions (‘confirmation’ step). In the age cohorts 12–< 18 years and 6–< 12 years, PK sampling also allowed for a non-compartmental analysis (NCA) of the raw data.

Subsequently (dashed lines in Fig. 2), the two rivaroxaban models were applied to simulate various dosing regimens for phase II and III studies, and PBPK and PopPK models were continuously validated and, if necessary, refined based on newly received data.

PBPK predictions for children

The full details of the pediatric PBPK model development process and the exposure predictions for rivaroxaban 10 mg per 70 kg and 20 mg per 70 kg have been presented previously [13]. In short, the starting point for the pediatric PBPK model was a rivaroxaban PBPK model for healthy adults that was built on and validated with rivaroxaban PK data observed at several dose levels in various phase I studies. The pediatric PBPK model was derived from this adult model via scaling of the physiological parameters to account for developmental changes that affect the PK of rivaroxaban. The model was then used to predict the PK behaviour of rivaroxaban in children prior to the start of any clinical study in pediatric subjects. These simulations showed a large overlap in PK parameters with values obtained for the corresponding doses in adults [13], and formed the basis for the body-weight-adjusted dosing scheme applied in phase I. Afterwards, PBPK model predictions were generated for each of the four age groups and two doses using the actual doses given. Each virtual age group consisted of 500 male children and 500 female children. The feeding status was randomly assigned as 50% fasted and 50% fed.

### Development of a first PopPK model in children

PopPK analyses in adults showed that the PK profile of rivaroxaban is appropriately described by a two-compartment model, provided that densely sampled concentration profiles are available to support the estimation of the parameters of the second compartment [20]. In adult patients, however, only a one-compartment model could be parameterised because of sparse blood sampling in phase II studies [21]. Therefore, a one-compartment model with exponential inter-individual variability (IIV) on the absorption rate constant (k_a_), clearance (CL) and volume of distribution (V) was chosen as the starting model for the analysis of the pediatric PK data. Initial model test runs demonstrated, however, that the data collected with the sparse sampling design (Fig. 1) were sufficient to inform a two-compartment model that could better fit the phase I pediatric data than a one-compartment model. Thus, the model structure was extended to a linear two-compartment model by introducing a peripheral compartment (Vp) that was coupled to the central compartmental via a first-order inter-compartmental clearance (Q). Absorption and elimination remained as first-order processes in the central compartment.

CL and V were allometrically scaled with body weight relative to a body weight of 70 kg to make the results similar to those from the PopPK studies performed in adults. Similar to observations in adults [21], the bioavailability was allowed to be different for the rivaroxaban 10 mg- and 20 mg-equivalent doses (with relative bioavailability defined as F1), and k_a_ was estimated separately for tablet, undiluted suspension and diluted suspension in order to account for potential effects of the formulation on the absorption rate.

### Data analysis and assessment of PBPK model predictivity

The concentration–time profiles predicted by the PBPK model per age cohort were overlaid with the corresponding observed data (using the actual sampling times for individual data points) to visually assess the validity of the PBPK model. Observed data were compared with the interval between the 5th and 95th percentiles of the PBPK prediction (90% prediction range) or an enlarged expected concentration range (representing 0.5-times 5th percentile and 1.5-times the 95th percentile of the PBPK prediction). The enlarged range was introduced to account for uncertainties in the estimation of some physiological parameters that may affect, for example, drug bioavailability and CL. The following PK parameters were derived from the PBPK predictions: area under the plasma concentration–time curve from time 0–24 h [AUC_0–24_], maximal plasma concentration [C_max_] and plasma concentration 24 h after rivaroxaban administration [C__24h_].

For children aged 6–18 years, the sparse sampling scheme allowed NCA of the raw data to calculate individual PK parameters (AUC_0–24_, C_max_ and C__24h_) using WinNonlin software (version 5.3; Pharsight Corporation, Mountain View, CA, USA) in conjunction with the Automation Extension 2.80 (Bayer AG, Berlin, Germany). However, because of the much lower temporal resolution of observations in the NCA, the definitions of C_max_ and C__24h_ different slightly compared with the PBPK and PopPK models. In the NCA, C_max_ denotes the maximum of all measured plasma concentrations per individual and C__24h_ is the concentration measured in the time interval 20–24 h after rivaroxaban administration. In contrast, the simulated C_max_, by definition, represents the absolute peak of the plasma concentration–time profile, which is likely higher than the observed maximum (because of the sparse sampling), and simulated C__24h_ is the concentration at exactly 24 h after dosing.

The analysis was conducted via non-linear mixed-effects modelling using NONMEM (ICON Development Solutions, Dublin, Ireland; version 7.2) with the Navigator workbench (Mango solutions, London, UK; version 9.2) on a Red Hat Enterprise Linux 6.3 environment. The first-order conditional estimation with interaction (FOCE with η-ε interaction) method was used. SAS (SAS institute Inc., Cary, NC, USA; version 9.2), R (The R Foundation for Statistical Computing, version 2.31) and PsN (version 3.4.1 with Perl version 5.10.1) were used for model evaluation and reporting.

## Results

From November 2010 to July 2015, a total of 59 children from 18 sites in seven countries (Australia, Austria, Canada, France, Israel, Italy and the United States) were enrolled and received rivaroxaban. Detailed demographic and baseline characteristics are published elsewhere [18]. All subjects were valid for PK analyses and contributed 206 plasma concentrations, of which seven were excluded because they were below the LLOQ.

### Observed plasma concentration–time data and PBPK model predictions

**Fig. 3:**
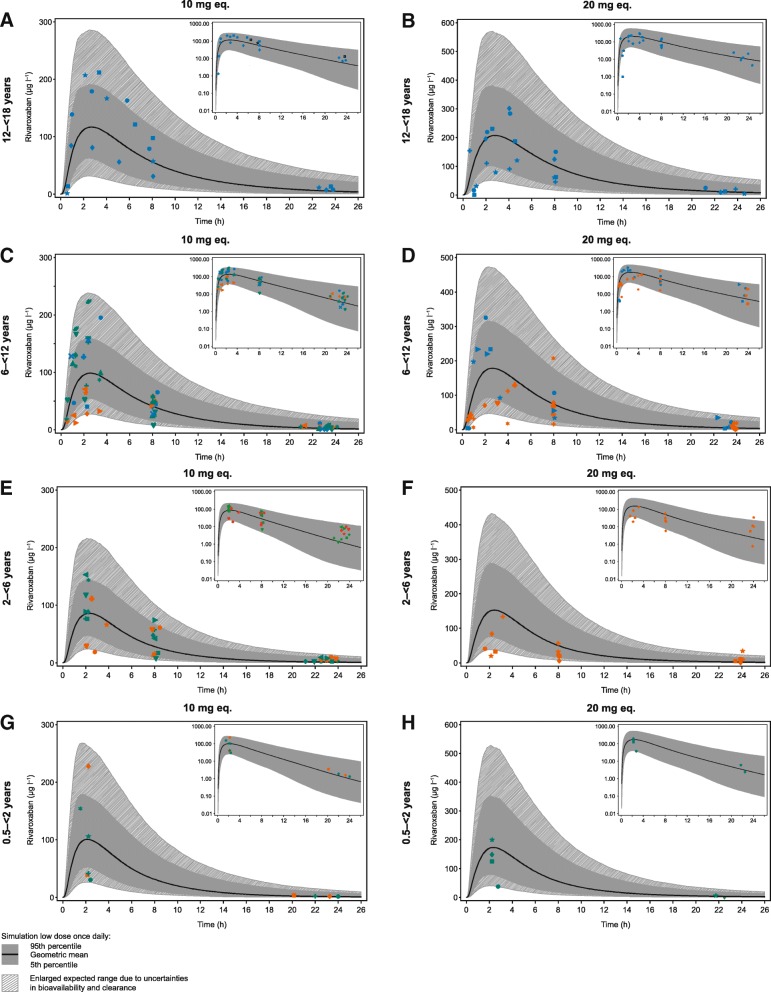
Plasma concentration–time curves in children aged **a** 12–18 years given 10 mg-equivalent rivaroxaban, **b** 12–18 years given 20 mg-equivalent rivaroxaban, **c** 6–12 years given 10 mg-equivalent rivaroxaban, **d** 6–12 years given 20 mg-equivalent rivaroxaban, **e** 2–6 years given 10 mg-equivalent rivaroxaban, **f** 2–6 years given 20 mg-equivalent rivaroxaban, **g** 0.5–2 years given 10 mg-equivalent rivaroxaban and **h** 0.5–2 years given 20 mg-equivalent rivaroxaban. The solid black line shows the geometric mean of the population prediction, the dark grey shaded area denotes the 90% prediction interval of the PBPK model. The light grey shading denotes the enlarged expected concentration range representing 0.5-times the 5th percentile and 1.5-times the 95th percentile of the PBPK prediction. Data points represent clinically observed data from individual subjects (blue: tablet formulation, orange: undiluted suspension, green: diluted suspension). The inset panels show the same data as the respective main panel on a semi-logarithmic concentration scale eq., equivalent; PBPK, physiologically based pharmacokinetic.

The observed plasma concentration–time data of the four age groups receiving the rivaroxaban 10 mg-equivalent and 20 mg-equivalent doses of rivaroxaban are shown in Fig. 3 in comparison with the PBPK model predictions.

#### Age group 12–18 years

Observed plasma concentration–time profiles of rivaroxaban 10 mg-equivalent and 20 mg-equivalent tablets were mostly within the 90% prediction interval (Fig. 3a and b). Only single data points in the early absorption phase were below the predicted range, indicating occasional cases of delayed absorption of the tablet formulation. However, the concentrations observed at later time points for these children are in good agreement with the PBPK predictions.

#### Age group 6–12 years

Eight children in this group received tablets, seven received diluted oral suspension and nine received undiluted oral suspension. Observed plasma concentrations with rivaroxaban 10 mg-equivalent or 20 mg-equivalent tablets were in good agreement with the PBPK predictions and, after completion of the absorption phase (i.e. < 4 h post administration), were close to the geometric mean of the PBPK prediction. Concentrations obtained during the absorption phase were rather variable but within the enlarged expected prediction range (Fig. 3c and d). Almost all observed concentration–time values with oral suspension of the rivaroxaban 10 mg-equivalent or 20 mg-equivalent doses were within the enlarged expected range of the PBPK model predictions (Fig. 3c and d). Subjects receiving the undiluted suspension of rivaroxaban 10 mg-equivalent dose displayed lower (close to or below the 5th percentile of the model prediction) plasma concentrations around the expected time to reach maximum plasma concentration (t_max_; 1–4 h after administration). However, plasma concentrations at approximately 8 h and between 20 and 24 h were close to the geometric mean of the PBPK prediction and similar to data obtained with tablets. Based on these observations, additional subjects received diluted rivaroxaban suspension at the 10 mg-equivalent and 20 mg-equivalent doses. The observed plasma concentration profiles with this diluted formulation were similar to profiles with the tablet formulation (Fig. 3c).

#### Age group 2–6 years

Of the four children who received undiluted suspension rivaroxaban 10 mg-equivalent, two had comparatively low plasma concentrations around the expected t_max_, and the other two showed concentration–time data that were well within the PBPK prediction range (Fig. 3e). Observed concentrations were close to the 95th percentile of the PBPK model prediction at 20–24 h after rivaroxaban administration. Seven additional children were enrolled to receive rivaroxaban 10 mg-equivalent as diluted suspension, and all their plasma concentrations around t_max_ were close to the geometric mean of the PBPK prediction (Fig. 3e). Five children received the rivaroxaban 20 mg-equivalent dose as undiluted suspension, four of whom had comparatively low plasma concentrations around the expected t_max_. Similar to older age groups, concentration values obtained at approximately 8 h were close to the PBPK-predicted geometric mean (Fig. 3f). Except in a single case, observed trough plasma concentrations were above the geometric mean of the PBPK prediction (Fig. 3f).

#### Age group 0.5–2 years

Figure 3g shows the plasma concentration–time data for the 10 mg-equivalent dose of rivaroxaban suspension compared with PBPK model predictions. Two children received the undiluted suspension, four received the diluted suspension and all displayed plasma concentration values within the enlarged expected range at t_max_; however, there was considerable IIV without any obvious difference in plasma concentration variability between the undiluted and diluted suspension. Four available plasma concentrations in the time interval 20–24 h were very close to the predicted geometric mean value. For two children, concentrations in this sampling interval were below the LLOQ. Four children received the rivaroxaban 20 mg-equivalent dose as diluted suspension (Fig. 3h). For three children, the plasma concentrations around the expected t_max_ were close to the geometric mean value of the PBPK predictions. For a single child, the concentration measured in the interval 1.5–5 h post administration was at the lower end of the enlarged expected range. This child received the diluted suspension via a baby bottle, but owing to a switch of the bottle, it is possible that the full dose was not received. At time points between 20 and 24 h after rivaroxaban administration, the plasma concentrations in two of the four children were close to the expected geometric mean value; in the other two children, the values were below the LLOQ.

### Parameters of the first PopPK model for rivaroxaban in children

The first attempt to develop a pediatric PopPK model for rivaroxaban resulted in a linear two-compartment model with first-order absorption and first-order elimination from the central compartment. The residual error was described by a proportional error model. The k_a_ was lower for the undiluted suspension in comparison to the tablet formulation and the diluted suspension (k_a_ was – according to the model – not different for the latter two formulations). The bioavailability of the rivaroxaban 20 mg-equivalent dose was approximately 65% of that of the rivaroxaban 10 mg-equivalent dose (which was set to 100% in this analysis). The scaling exponents of V with body weight was estimated to be not significantly different from 1; therefore, it was fixed to 1, consistent with the allometric theory. Scaling of Vp and Q with body weight did not improve the fit and led to implausibly large values of Vp. For CL, an allometric exponent of 0.323 was estimated, which was lower than the value expected from the allometric theory (0.75).

The parameters of the first pediatric PopPK model for rivaroxaban are summarised in Table 1. The majority of the estimated standard errors were well below 50%, with the exception of Vp (52%) and IIV on k_a_ (64%). The degree of η-shrinkage was 17% for CL and 37% for k_a_, which was slightly above the widely accepted threshold of 30%. Such a degree of shrinkage was not unexpected given the sparseness of the analysed data. Consequently, the distribution of all post hoc parameters obtained with this model will likely be smaller than the real distribution.

**Table 1:** Population estimates for the first pediatric PopPK model of rivaroxaban

Parameter	Mean estimate	Relative standard error (%)^a^	Inter-individual variability CV (%)^b^	Relative standard error (%)^†^	Description
k_a_ for tablet and diluted suspension (1 h^− 1^)	0.717	21.3	39.7	63.9	Absorption rate constant
k_a_ for undiluted suspension (1 h^−1^)	0.208	15.4			
CL (l h^−1^)	7.26	9.38	26.2	39.2	Clearance for a subject with a body weight of 70 kg
V (l)	50.9	12	N/A	N/A	Volume of distribution in the central compartment for a subject with a body weight of 70 kg
Q (l h^− 1^)	0.928	17.5	N/A	N/A	Intercompartmental clearance
Vp (l)	13.5	51.5	N/A	N/A	Volume of distribution in the peripheral compartment
Exponent for CL scaling	0.323	27.1	–	–	Exponent to scale CL on the individual body weight
Exponent for V scaling	1	Fixed	–	–	Exponent to scale V on the individual body weight
F1	0.648	9.03	N/A	N/A	Relative bioavailability for the rivaroxaban 20 mg-equivalent doses in relation to the rivaroxaban 10 mg-equivalent doses with F1 = 1 per definition
Residual error (%)	46.6	14.1	–	–	Proportional residual error

^a^Relative standard error expressed as a percentage of the estimate; ^b^coefficient of variation, calculated as the square root of the variance (which is approximately equivalent to coefficient of variation [%])

*CL*, clearance, *CV* coefficient of variation, *k*_*a*_ absorption rate constant, *PopPK* population pharmacokinetic, *Q* first-order inter-compartmental clearance, *V* volume of distribution *Vp* peripheral compartment

### Comparison of PK parameters with PBPK model predictions

AUC_0–24_, C_max_ and C__24h_ were estimated using the PopPK model for all 59 children and NCA was performed in all 33 children ≥6 years of age (Table 2). NCA-derived PK parameters were similar to the corresponding parameters obtained from the PopPK model.

**Table 2:** PK parameters of rivaroxaban derived via NCA (12–18 years and 6–12 years) or PopPK analysis (geometric mean/CV [range])

		12–18 years		6–12 years				2–6 years		0.5–2 years	
		Tablet rivaroxaban 10 mg eq. (*n* = 4)	Table trivaroxaban 20 mg eq. (*n* = 5)	Tablet rivaroxaban 10 mg eq. (*n* = 4)	Suspension rivaroxaban 10 mg eq. (*n* = 11)	Tablet rivaroxaban 20 mg eq. (NCA *n* = 4, PopPK *n* = 5)	Suspension rivaroxaban 20 mg eq. (*n* = 5)	Suspension rivaroxaban 10 mg eq. (*n* = 11)	Suspension rivaroxaban 20 mg eq. (*n* = 5)	Suspension rivaroxaban 10 mg eq. (*n* = 6)	Suspension rivaroxaban 20 mg eq. (*n* = 4)
AUC_0–24_ (μg•h l^−1^)	PopPK	1320/13.1 (1090–1450)	1760/20.7 (1270–2090)	902/31.2 (645–1340)	720/24.1 (443–1120)	1540/52.0 (917–2390)	1070/37.5 (784–1700)	674/25.5 (433–976)	755/39.0 (476–1110)	503/20.6 (373–660)	672/29.4 (461–929)
	NCA	1340/37.7 (788–1760)	1660/39.0 (968–2560)	866/36.0 (614–1410)	719/42.4 (293–1380)	1650/38.9 (1050–2410)	1060/48.0 (572–1890)				
C_max_ (μg l^−1^)	PopPK	129/11.4 (115–143)	180/12.0 (148–200)	118/11.0 (105–136)	82.5/37.4 (47.2–137)	172/25.9 (128–218)	85.1/15.7 (72.1–106)	84.2/36.0 (46.7–132)	60.1/33.4 (44.9–92.6)	94.9/28.3 (56.0–125)	143/14.7 (118–169)
	NCA	161/45.6 (84.5–213)	206/41.3 (121–302)	115/72.6 (44.9–195)	89.2/59.3 (42.0–224)	244/21.1 (198–325)	113/42.9 (77.0–208)				
C__24h_^a^ (μg l^− 1^)	PopPK	7.94/35.4 (5.25–12.2)	9.46/55.6 (4.42–16.5)	3.18/73.9 (1.71–7.52)	3.35/57.6 (0.873–5.69)	8.46/98.5 (3.45–19.0)	7.18/96.1 (3.27–18.0)	3.27/65.0 (1.11–7.61)	5.71/129 (1.25–18.2)	1.94/33.5 (1.19–2.87)	2.39/39.9 (1.56–3.95)
	NCA	9.55/30.0 (7.10–13.3)	11.9/77.0 (4.47–24.3)	3.43/127 (1.27–11.2)	4.18/64.0 (0.952–8.20)	12.5/128 (3.87–35.4)	7.44/123 (2.81–19.2)				

^a^C__24h_ derived via NCA here indicates the concentration obtained in the last sampling interval, i.e. 20–24 h after rivaroxaban administration

AUC_0–24_, area under the plasma concentration–time curve from time 0–24 h; C_max_, maximal plasma concentration; NCA, non-compartmental analysis; C__24h_, plasma concentration 24 h after rivaroxaban administration; eq., equivalent; PopPK, population pharmacokinetic

**Fig. 4:**
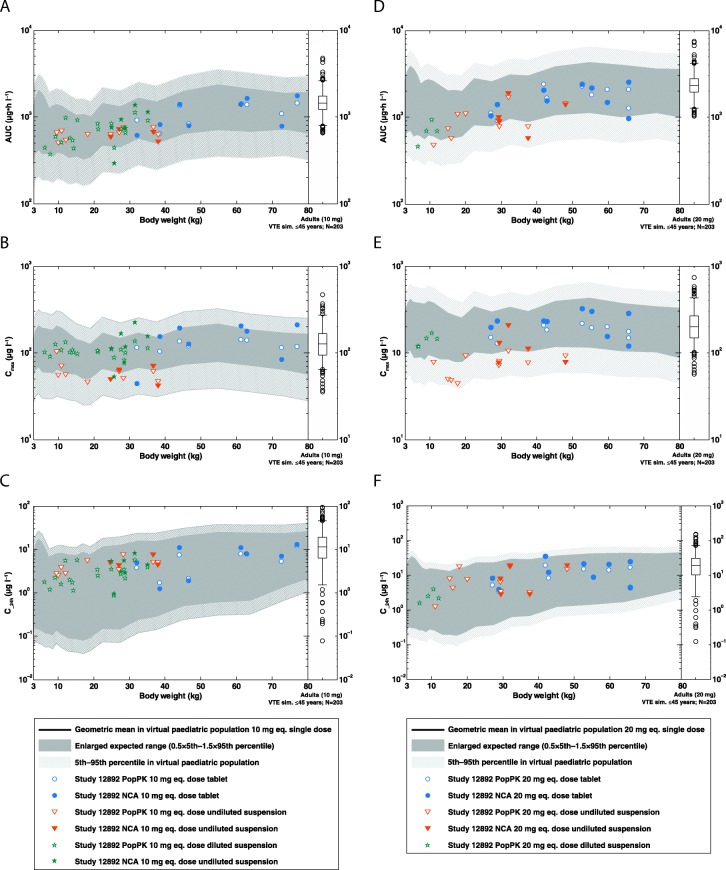
Range plots comparing PK parameters for children aged 0.5–18 years derived from the PopPK analysis or NCA (aged ≥6 years) with the corresponding PBPK model predictions: **a** AUC_0–24_, **b** C_max_ and **c** C__24h_ for rivaroxaban 10 mg-equivalent doses, and **d** AUC_0–24_, **e** C_max_ and **f** C__24h_ for rivaroxaban 20 mg-equivalent doses. The solid black line shows the geometric mean of the population prediction and the light grey shaded area denotes the 90% prediction interval of the PBPK model. The dark grey shading denotes the enlarged expected concentration range representing 0.5-times the 5th percentile and 1.5-times the 95th percentile of the PBPK prediction. Data points show PK parameters of individual subjects derived by NCA (closed symbols) or PopPK analysis (open symbols). The corresponding distributions of PK parameters observed via PopPK modelling from an adult reference population (*N* = 203 adult VTE patients aged 18–45 years) is also shown as box-whisker plot indicating the percentiles 5, 25, 50, 75 and 95 AUC_0–24_, area under the plasma concentration–time curve from time 0–24 h; C__24h_, plasma concentration 24 h after rivaroxaban administration; C_max_, maximal plasma concentration; eq., equivalent; NCA, non-compartmental analysis; PBPK, physiologically based pharmacokinetics; PK, pharmacokinetics; PopPK, population pharmacokinetic; VTE, venous thromboembolism.

Figure 4 shows body-weight-dependent AUC_0–24_, C_max_ and C__24h_ and compares the predictions for children and adolescents based on the PBPK model with the individual PK parameters derived from the observed data using either PopPK analysis or NCA. In addition, the pediatric PK parameters were compared to adult reference values based on phase II dose-ranging studies [22, 23]. In younger children, there is a tendency towards lower exposures, which is in line with the pediatric PBPK predictions due to the cautious dosing approach. All PopPK and NCA-derived values for AUC_0–24_, C_max_ and C__24h_ for both doses were within the enlarged expected ranges of the PBPK predictions.

## Discussion

In this study, children received body-weight-adjusted doses of rivaroxaban that were derived from dose–exposure relations predicted by PBPK modelling [13]. The results demonstrated the applicability of the rivaroxaban pediatric PBPK model as an appropriate basis for informing dose selection for children aged 0.5–18 years in future pediatric VTE studies of the EINSTEIN-Jr program and confirmed the applicability of the ‘predict-learn-confirm’ approach using PBPK modelling in children.

During the study, PK data were periodically evaluated to check whether the results were in line with the PBPK model predictions. For all age groups and irrespective of dose or formulation, nearly all observed plasma concentration–time values after single doses of rivaroxaban of either 10 mg-equivalent or 20 mg-equivalent were within the enlarged expected ranges (Fig. 3). However, for four children aged 0.5–< 2 years rivaroxaban plasma concentrations were below the LLOQ in the interval 20–24 h post dose and, thus, an interpretation was not possible for these children (Fig. 3g, h).

For the tablet formulation, most concentration values were close to the geometric mean of the PBPK model prediction after termination of the absorption phase (Fig. 3a-d). PK data with the undiluted suspension in the 6–12 years group demonstrated a delayed absorption compared with the tablet formulation (Fig. 3c, d). Based on subsequent in vitro dissolution data (data not shown) it was concluded that the delay in absorption could have been caused by excipients of the oral suspension which limit dissolution of rivaroxaban particles at low pH. The in vitro results further indicated that dilution of the oral suspension may overcome this delayed absorption, which was subsequently confirmed in vivo. In children aged 6–12 years who received diluted suspension, no delay in the absorption profile was seen (Fig. 3c, d). In the youngest age group (0.5–2 years), significant IIV was observed around t_max_ independent of the use of the undiluted or diluted suspension (Fig. 3g, h).

The influence of the formulation on the PK of rivaroxaban was also assessed in the PopPK analysis. In line with the observations, the PopPK model estimated that the k_a_ for the undiluted suspension would be lower than the absorption rate of the tablet or diluted suspension, for which no difference was found. Bioavailability was not affected by the formulation or dissolution process, but by dose level. The relative bioavailability between the rivaroxaban 10 mg-equivalent and 20 mg-equivalent doses was estimated to be approximately 65%, which was consistent with adult studies that showed a relative bioavailability of 79% of the 20 mg regimen compared with the 10 mg regimen [21]. Because no intravenous data in children are available, the absolute bioavailabilities of the rivaroxaban 10 mg-equivalent and 20 mg-equivalent doses remain unknown, but the PBPK model suggested that absorption of the 10 mg-equivalent dose is nearly complete in all children.

This model was the first PopPK model for rivaroxaban in children. Although sampling was sparse, the complete dataset was best described by a linear two-compartment model. CL and V were scaled by body weight with exponents of 1 (V) and 0.323 (CL). The exponent for CL was considerably smaller than expected from the allometric theory (0.75), which was possibly due to the relatively small number of young children. IIV could be quantified for CL and k_a_; the estimates of IIV on CL and KA were well in line with those established in adults [20]. The residual error was described by a proportional error model. Although the majority of estimated standard errors were well below 50%, the IIV on k_a_ was 64%, indicating a potential imprecision in the description of the absorption phase of this model.

The PopPK model was used to obtain individual post hoc estimates for AUC_0–24_, C_max_ and C__24h_, which were then compared with NCA-derived PK parameters in children aged ≥6 years and PBPK predictions of the virtual pediatric populations. The NCA-derived PK parameters were similar to the corresponding parameters obtained from the PopPK model. For C__24h_, the NCA-derived geometric mean was consistently larger in all cohorts compared with the PopPK estimate, which was not unexpected because the NCA value denotes the minimal observed concentration in the sampling time window of 20–24 h post rivaroxaban dose, whereas in the PopPK analysis C__24h_ was defined as the concentration at exactly 24 h.

All derived PK parameters for AUC_0–24_, C_max_ and C__24h_ were in good agreement with the PBPK predictions, because all individual values were within the predicted enlarged expected ranges (Fig. 4). There was no systematic trend towards either under- or overprediction of any of these PK parameters, with the exception of C_max_ with undiluted suspension, which tended to be at the lower end of the prediction ranges. Additionally, the observed IIV in the NCA-derived PK parameters was also in line with the expected ranges based on PBPK modelling. For the post hoc estimates derived via PopPK modelling, it has to be noted that because of the small number of subjects and sparse data collected, the PopPK model was considerably affected by a phenomenon called ‘η-shrinkage’, i.e. the individual post hoc estimates had a tendency towards the population mean and, consequently, did not display the true IIV.

Our results confirmed that the PBPK model can closely predict the PK of rivaroxaban in children as young as 0.5 years. Together with the first pediatric PopPK model for rivaroxaban, there are now two reliable modelling and simulation approaches available to support the development of rivaroxaban in the EINSTEIN-Jr program. Additionally, this study also demonstrates the applicability of the ‘predict-learn-confirm’ approach using PBPK and PopPK modelling in pediatric drug development.

We do not report the identified rivaroxaban dose regimens because the results from the combination of the rivaroxaban pediatric PBPK and PopPK models will only be used to guide dose selection and, therefore, there is the potential that further changes in dose regimens will be implemented based on the EINSTEIN-Jr phase II and III studies. Off-label use of preliminary rivaroxaban dose regimens in children could put them at risk of thrombotic or bleeding complications.

## Conclusions

Our results confirmed the applicability of the rivaroxaban pediatric PBPK model, which in combination with the PopPK model, will be used to further guide dose selection for the treatment of VTE with rivaroxaban in the EINSTEIN-Jr phase II and III studies.

## Abbreviations

AUC_0–24_: Area under the plasma concentration–time curve from time 0–24 h
C_24h: Plasma concentration 24 h after rivaroxaban administration
CL: Clearance
C_max_: Maximal plasma concentration
IIV: Exponential inter-individual variability
Ka: Absorption rate constant
LLOQ: Lower limit of quantification
NCA: Non-compartmental analysis
PBPK Model: Physiologically based pharmacokinetic model
PD: Pharmacodynamic
PK: Pharmacokinetics
PopPK: Population pharmacokinetic
Q: Inter-compartmental clearance
t_max_: Maximum plasma concentration
V: Volume of distribution
Vp: Peripheral compartment
VTE: Venous thromboembolism
